# Supplementary figures and images for: SARS-CoV-2 ORF3A interacts with the Clic-like chloride channel-1 (CLCC1) and triggers an unfolded protein response
Source: PeerJ. 2023 Apr 3;11:e15077. doi: 10.7717/peerj.15077 (PMC10078464; doi:10.7717/peerj.15077)

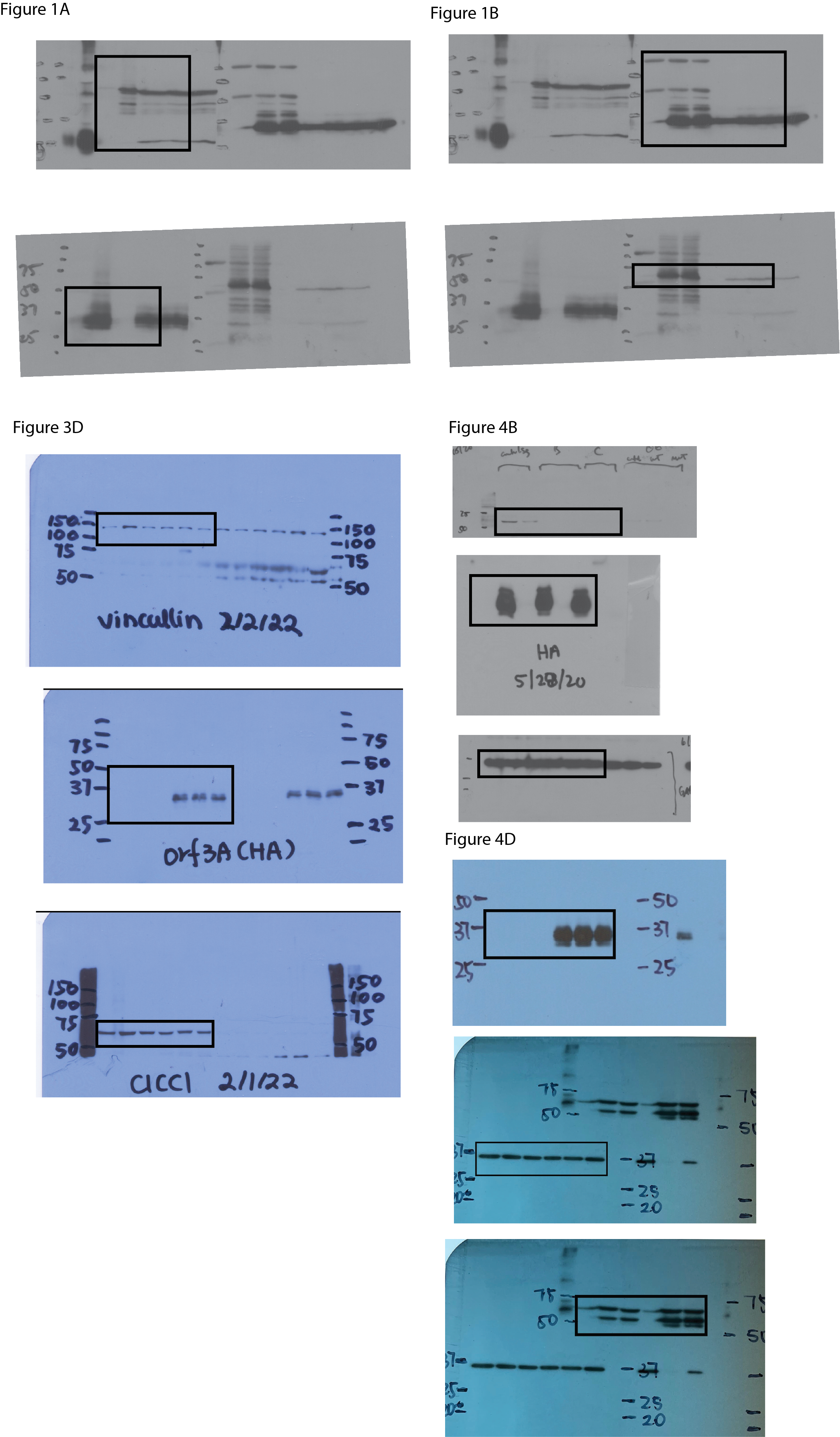

Supplement: Supplemental Information 1 [file peerj-11-15077-s004.png]
